# The cocktail-party problem revisited: early processing and selection of multi-talker speech

**DOI:** 10.3758/s13414-015-0882-9

**Published:** 2015-04-01

**Authors:** Adelbert W. Bronkhorst

**Affiliations:** 1 TNO Human Factors, POB 23, 3769 ZG Soesterberg, The Netherlands; 2 Department of Cognitive Psychology, Vrije Universiteit, van den Boechorststraat 1, 1081 BT Amsterdam, The Netherlands

**Keywords:** Attention, Auditory scene analysis, Cocktail-party problem, Informational masking, Speech perception

## Abstract

How do we recognize what one person is saying when others are speaking at the same time? This review summarizes widespread research in psychoacoustics, auditory scene analysis, and attention, all dealing with early processing and selection of speech, which has been stimulated by this question. Important effects occurring at the peripheral and brainstem levels are mutual masking of sounds and “unmasking” resulting from binaural listening. Psychoacoustic models have been developed that can predict these effects accurately, albeit using computational approaches rather than approximations of neural processing. Grouping—the segregation and streaming of sounds—represents a subsequent processing stage that interacts closely with attention. Sounds can be easily grouped—and subsequently selected—using primitive features such as spatial location and fundamental frequency. More complex processing is required when lexical, syntactic, or semantic information is used. Whereas it is now clear that such processing can take place preattentively, there also is evidence that the processing depth depends on the task-relevancy of the sound. This is consistent with the presence of a feedback loop in attentional control, triggering enhancement of to-be-selected input. Despite recent progress, there are still many unresolved issues: there is a need for integrative models that are neurophysiologically plausible, for research into grouping based on other than spatial or voice-related cues, for studies explicitly addressing endogenous and exogenous attention, for an explanation of the remarkable sluggishness of attention focused on dynamically changing sounds, and for research elucidating the distinction between binaural speech perception and sound localization.

Speech communication is so all-pervasive and natural that it is easy to underestimate the formidable difficulties our auditory system has to overcome to be able to extract meaningful information from the complex auditory signals entering our ears. In particular in environments where we try to understand one talker among multiple persons speaking at the same time, the capacities of the auditory system are stretched to the limit. To most of us blessed with normal hearing, it seems as if this task is achieved without any effort, but the fragility of speech perception is clearly revealed when there is background noise or when a hearing impairment affects the peripheral encoding of the incoming signals. The difficulties associated with understanding speech in multiple-talker situations often are associated with the term “cocktail-party problem” (or “cocktail-party effect”), coined by Colin Cherry in his 1953 paper. While the widespread use of this term might suggest the existence of a single, coherent field of research, scientific work has actually for many years proceeded along different lines that showed little or no overlap. Cherry himself was mainly interested in the ability of listeners to select target speech while ignoring other sounds in conditions where signals were either mixed or presented to separate ears. This work acted as starting point of a line of research into selective attention, which generated influential early “filter” models (Broadbent, 1958; Treisman, 1964; Deutsch & Deutsch, 1963). Later work on attention has predominantly focused on the visual modality and it was not until relatively recently that further progress was made in understanding how auditory attention affects speech perception (Cowan & Wood, 1997; Pulvermüller & Shtyrov, 2006; Parmentier, 2013).

A line of research with an even longer history has studied how simultaneous sounds interfere with each other already at the peripheral level. It originated at Bell Labs in the beginning of the previous century (Allen, 1994) and has culminated in the development of powerful models that can predict effects of various interfering sounds on speech intelligibility (French & Steinberg, 1947; Jørgensen, Ewert, & Dau, 2013). An important finding, which is incorporated in more recent models and which is relevant for “cocktail-party” conditions, is that the auditory system benefits considerably from the fact that we have two ears. The head provides an acoustic “shadow,” which can favor one ear, depending on the location of the talkers. In addition, the differences between the signals entering the two ears enable us to partially “unmask” interfering sounds, effectively providing an increase of the signal-to-noise ratio (SNR: the ratio of levels of target and interfering sounds) of up to 4 dB (Bronkhorst & Plomp, 1988).

While it is evident that speech must be audible and needs to be selected in order to be understood, there is actually a third crucial stage in the early processing of speech, which is addressed in the third line of research reviewed here. In this stage, individual speech elements are grouped together into streams. Past research into selection and audibility did not take this into account because it used stimuli that can be easily grouped, e.g., sounds presented to the two ears or speech mixed with interfering noise. An early review of research on grouping was written by Bregman (1990), who then had to rely mainly on results from experiments conducted with non-speech stimuli. Fortunately, grouping of speech sounds was addressed in many later studies, in particular those investigating “informational masking”: interference that cannot be explained by reduced audibility (Brungart, 2001; Arbogast, Mason, & Kidd, 2002). Bregman’s (1990) review revived interest in the “cocktail party” effect and introduced a novel term for the research area—auditory scene analysis—that has been widely adopted. However, because this term also refers to attentional effects, it does not fit into the distinction between research lines made in this review. Thus, the term “grouping” is used instead.

This review is intended to supplement an earlier one (Bronkhorst, 2000), which mainly considered the second of the three research lines. Its purpose is to discuss all lines and integrate the results in a single framework. This task is facilitated by the increasing number of studies that cross the “boundaries” of the research lines. The work on informational masking provides a good example, because it looks at effects of attention and/or grouping while controlling for audibility (Gallun, Mason, & Kidd, 2005; Freyman, Balakrishnan, & Helfer, 2001). The review restricts itself to the three research lines and to early processing of speech by normal-hearing listeners, which means that it does not include animal research or work on psycholinguistics, memory, or hearing impairment. It focuses on studies that use speech stimuli, but incidentally, for example when there is a lack of data, results for non-speech stimuli are considered as well. The organization of the review is as follows. After a short section that considers speech itself, there are sections addressing the three research lines. In the sixth section, a conceptual, integrative model of auditory processing of multi-talker speech is presented. The review ends with suggestions for future research. It is important to acknowledge that this review has been inspired by earlier overviews published by others, in particular the work of Bregman (1990) and more recent reviews written by Darwin (1997; 2008), Assman and Summerfield (2004), Shinn-Cunningham (2008), McDermott (2009), Näätänen, Kujala, and Winkler (2011), and Moore and Gockel (2012).

## How “special” is speech?

If engineers would design an acoustic signal for communication among humans, resistant to all kinds of acoustic interferences, they would probably not come up with something resembling natural speech. With its voiced phonemes that show large variations in fundamental frequency (F0) across talkers, rapidly alternating with unvoiced phonemes that range from noise-like sounds to stops, it seems an unlikely candidate. Speech, however, appears to be remarkably well suited for its purpose. Acoustic analyses and vocal tract modeling show that phonemes (a) are relatively invariant to vocal tract differences, (b) make good use of the available “perceptual space” (a simple space for vowels can be defined by the frequencies of the first two resonance peaks, or formants), and, by concentrating energy in limited spectral regions, (c) are resistant to masking by background noise (Diehl, 2008). Furthermore, the information contained in speech is coded with such redundancy that (d) missing parts often can be “reconstructed.”

The latter two properties are particularly relevant in “cocktail-party” conditions. The fact that speech energy is concentrated in discrete spectrotemporal regions has as consequence that, when speech is mixed with interfering speech or other sounds, it, on average, will still deliver the dominant contribution to many regions. This is enhanced by the fact, noted by Darwin (2008), that due to the logarithmic intensity transformation performed by the auditory system, the “winner takes all” when a stronger signal is added to a weaker one. An interesting application of this effect is the use of binary masks in automatic speech segregation that attempt to identify and remove all spectrotemporal regions that are not dominated by the target speech (Roman, Wang & Brown, 2003; Hu & Wang, 2004; Cooke, 2006).

The redundancy of speech is apparent from its resistance against various kinds of distortions, such as bandwidth reduction (French & Steinberg, 1947), peak clipping (Pollack & Pickett, 1959), temporal smearing (Drullman et al. 1993), and even changes in the carrier to which spectrotemporal modulations are applied (Remez, Rubin, Pisoni, & Carell, 1981). There are many types of redundancies, ranging from acoustic effects at the phonetic level (e.g., coarticulation between phonemes) to contextual information at the sentence level (Kalikow, Stevens, & Elliot, 1977; Boothroyd & Nittrouer, 1988). Given that redundancy acts as a kind of “safety net” that allows missing information to be recovered, its effects can be quantified by determining how well listeners can fill in missing speech parts. Whereas these “cloze probabilities” can be measured directly with written text (Taylor, 1953; Block & Baldwin, 2010), an indirect method has to be used for speech because removing phonemes or words will almost always disrupt coarticulatory cues and/or introduce false cues. Bronkhorst, Bosman, and Smoorenburg (1993) and Bronkhorst, Brand, and Wegener (2002) developed such a method, which is based on probabilities of occurrence of correct and partially correct responses. They estimated that, for speech masked by noise, the probability of recovering a missing phoneme is approximately 50 % for meaningful words and 20 % for nonsense words. Words missing from short everyday sentences can be filled in more easily with 70–90 % accuracy, depending on sentence length. For meaningless sentences with a known syntactic structure, the probability is still approximately 50 %.^1^ These data not only show that there is more contextual information on the word than on the phoneme level (as expected), but also that there is a major contribution of nonsemantic information.

Thus, speech is certainly “special,” not only because it satisfies several acoustic criteria, but also because we are finely tuned to its properties. Studies of speech intelligibility in noise for nonnative listeners demonstrate that this tuning requires exposure early in life. Mayo, Florentine, and Buus (1997), for example, compared performance of native listeners with that of early bilinguals, who learned a second language before age 6 years, and late bilinguals, learning it after age 14 years. They found similar SRTs (Speech Reception Thresholds: SNRs required for 50 % sentence intelligibility) for the first two groups, but 4–5 dB higher SRTs for the late bilinguals. This is a considerable penalty-expressed in average speech levels in “cocktail party” conditions this is equivalent to a threefold increase of the number of interfering talkers. The fact that tuning occurs does not necessarily imply that the processing of speech by the auditory system is entirely different from that of other sounds. On the one hand, EEG and neuroimaging studies indicate that there are specialized brain regions for speech processing (Patterson & Johnsrude, 2008) and that the infant brain already responds differently to native speech than to other speech or to nonspeech sounds (Kuhl & Rivera-Gaxiola, 2008). On the other hand, there are remarkable similarities in neural processing of speech by animals and humans (Steinschneider, Nourski, & Fishman, 2013) and it also seems unlikely that low-level preattentive processing uses separate mechanisms for speech and other sounds (Darwin, 2008).

## Masking and Unmasking of Speech Sounds

Normally, sounds originating from different sources will always reach both ears, which means that there will be interference between incoming sounds already at a peripheral level. This peripheral interference is called *masking* or to distinguish it from informational masking: *energetic masking*. Our hearing system also benefits from the fact that interfering signals reaching the two ears have similar components, because it can suppress such components and thus achieve an effective reduction of masking, referred to as *unmasking*. When studying masking, a crucial variable is the SNR. As shown by Plomp (1977), SNRs in a typical cocktail party are in theory around 0 dB, which would mean that sentences can be easily understood, whereas isolated words are somewhat less intelligible. In practice, however, interfering sound levels often will be higher (e.g., because of background noise) and listeners will perform less then optimally (e.g., because of a hearing impairment). This means that the gain afforded by binaural listening can be crucial to reach sufficient intelligibility levels.

Many factors that influence (un)masking of speech have been discussed by Bronkhorst (2000); the most relevant are the type of target speech, spectral differences between target and interfering sounds, the spatial configuration of the sound sources, fluctuations in level (modulations) of the interfering sounds, the acoustics of the environment, and hearing impairment of the listener. The effects of these factors often are quantified as shifts of the SRT. For example (see also Table 1 in Bronkhorst, 2000), spatial separation of sound sources and level fluctuations of interfering sounds can each cause positive effects (SRT reductions) of up to 10 dB. Spectral differences will have smaller positive effects, of up to 5 dB. Reverberation and (moderate) hearing impairment can result in large negative effects of up to 10 dB. In view of the number of factors, and given that some of them interact with each other, it is clear that one needs models encompassing as many factors as possible when trying to make sense of the experimental data. I will, therefore, concentrate on the evolution of the most important speech perception models, summarizing their main properties and indicating their potential and limitations. An overview of the models that are discussed and their main features is given in Table 1.

**Table 1:** Overview of monaural and binaural speech perception models

Model for quantifying speech information		References	Aspects that are modeled	Binaural model	References (binaural version)
Speech Intelligibility Index (SII)		a	I, B, F (refs. f, i)	Equalization Cancellation	g, h, i, j
Speech Transmission Index (STI)		b	I, B, R, T	Binaural version of STI	k
Speech-based Envelope Power Spectrum Model (sEPSM)		c, d	I, B, F, R, T, P		
Speech Recognition Sensitivity Model (SRS)		e	I, B, P		
(No model)				Descriptive model of binaural gain	l, m
References	a	ANSI, 1997			
	b	IEC, 2003			
	c, d	Jørgensen & Dau, 2011; Jørgensen et al. (2013)			
	e	Müsch and Buus (2001)			
	f	Rhebergen et al. (2006)			
	g	Durlach (1972)			
	h, i, j	Wan et al. (2010), Beutelmann et al.(2010), Lavandier et al. (2012)			
	k	Van Wijngaarden and Drullman (2008)			
	l, m	Bronkhorst (2000), Jones and Litovsky (2011)			
Aspects that are modeled	I	Long-term average frequency spectra of speech and interference			
	B	Bandwidth reduction of speech and/or interference			
	F	Envelope fluctuations of the interference			
	R	Effect of reverberation on target speech			
	T	Time-domain distortions of target speech (e.g. peak clipping)			
	P	Implicit modeling of the psychometric function			

### Quantifying speech information and effects of interfering noise

A fundamental property of any model predicting speech intelligibility is the way in which speech information is quantified. The predictor with the longest history is the Speech Intelligibility Index (SII; ANSI, 1997), originally called the Articulation Index (AI; French & Steinberg, 1947; Kryter, 1962). Basically, the SII is determined by calculating SNRs in nonoverlapping frequency bands, truncating these to the range −15 to +15 dB, mapping them linearly to values between 0 and the value of the “importance function” for that band, and finally summing them across bands.^2^ The SII is widely used and has been extensively validated. Advantages are that frequency-domain effects, in particular differences in long-term average frequency spectra of target and interfering sounds, are modeled quite accurately and that, in contrast to percent correct scores that are difficult to compare across experiments, the index represents a generic, uniform measure of speech intelligibility. An important disadvantage is, however, that differences in speech material affect the model at two stages: the band importance function used to calculate the index, and the psychometric function used to map index values to percent correct values. This means that it is actually not easy to adapt the SII to different types of speech. Other shortcomings are that effects of reverberation and of interferer modulations are not modeled. As shown by Rhebergen and Versfeld (2005), the latter factor can actually be approximated relatively easily by calculating the SII in short time frames (varying from 9.4 to 35 ms, depending on frequency) and then averaging it over time. The accuracy of the predictions can be improved by taking forward masking into account; in that case, a constant time frame of 4 ms can be used (Rhebergen, Versfeld & Dreschler, 2006).

Another widely used predictor is the Speech Transmission Index (IEC, 2003; Steeneken & Houtgast, 1980). This index borrows the SNR-based approach from the SII/AI to model frequency-domain masking effects but uses preservation of speech modulations as a measure for quantifying time-domain distortions, such as reverberation and peak clipping. The STI, in effect, quantifies how speech quality deteriorates when it is transmitted through an electric and/or acoustic channel. The crucial parameter is the modulation transfer function (MTF)—the quotient of the modulation depths at the output and input of the channel. The MTF is determined in frequency bands, converted to equivalent SNRs using the equation SNR = 10log(*MTF*/(1-*MTF*)) and then weighted with a band importance function in a similar way as is done in the SII calculation. Recently, this modulation-based approach has been generalized to a “speech-based envelope power spectrum model” (sEPSM; Jørgensen & Dau, 2011). Instead of using SNRs based on frequency spectra, this model uses SNRs in the envelope power domain, derived from the power of the envelopes of the noise and speech + noise signals. This model adds complexity to the STI, because it determines these SNRs in modulation bands as well as frequency bands. However, because it converts SNRs to *d*’ values and then uses an ideal observer to calculate percent correct values, it not only implicitly models the psychometric function, but also includes the effect of response set size (i.e., the performance increase related to reduction of the number of response alternatives). Another crucial difference with the STI and SII approaches is that no band importance function is used to weigh contributions of frequency bands. Differences in speech material are accounted for by adjusting four parameters: two used in the conversion of SNRs to *d*’ values, and two (one is the response set size) entering the calculation of percentage correct scores. Jørgensen et al. (2013) recently developed a multiresolution version of this model to be able to predict effects of interferer modulations as well. The method used is roughly similar to that of Rhebergen and Versfeld (2005), discussed above. The averaging across time frames is, however, done with envelope power SNRs and time frames have durations depending on the modulation band.

A third approach to predicting speech perception was introduced by Müsch and Buus (2001). Their Speech Recognition Sensitivity (SRS) model is in essence a model of speech transmission, just as the STI and the sEPSM are, because it quantifies speech degradation. Different types of degradation are modeled as independent sources of variance that affect the match between an ideal speech signal and the template of that signal used by the listener to identify it. The degradations are imperfections in speech production, interfering sounds, and “cognitive noise,” representing speech entropy determined by various contextual cues (Van Rooij & Plomp, 1991). The model generates a *d*’ value just as the sEPSM model does. A specific feature is that intelligibility of speech presented in spectrally disjoint frequency bands can be predicted by taking into account synergetic effects, which are not modeled in the SII and STI approaches. The model, however, requires many parameters and is, as yet, not able to predict effects of reverberation and interferer modulations.

It should be noted that there are constraints limiting how accurately speech information can be quantified, because speech intelligibility depends on many properties of the speech material—in particular its linguistic, syntactic, and semantic information—and on possible interactions with interfering sounds. All models presented above can be adapted to some degree to such variations, but this is normally done in a relatively coarse way, for example based on corpora consisting of certain types of sentences or words, in combination with specific types of interfering sounds (e.g., IEC, 2003, Fig. 1). However, there may be considerable differences in intelligibility between items in a corpus. Van Rooij and Plomp (1991), for example, tested the sentence set developed by Plomp and Mimpen (1979), designed to be relatively homogeneous, and found differences of up to 4 dB in the SRT in noise between individual sentences. That speech material and type of interference can interact with each other was recently demonstrated by Uslar, Carroll, Hanke, Hamann, Ruigendijk et al. (2013), who found that variations in the linguistic complexity of speech material affected intelligibility differently for steady-state than for fluctuating interfering noise.

### Binaural speech perception

The models described above can account for effects of interfering sounds and reverberation but do not predict the gain resulting from binaural listening. Three types of cues should be taken into account to achieve this: interaural time differences (ITDs, differences in arrival time between the ears), interaural level differences (ILDs), and interaural decorrelation (reduced coherence). The latter factor, which occurs in any environment where there is reverberation, results from differences between the reflections arriving at the two ears (Hartmann, Rakerd & Koller, 2005). The binaural cues depend on many acoustic factors: the spatial configuration and directivity of the sound sources, the room geometry and reverberation, and the shape of the head and ears of the listener. As a result, they cannot be calculated easily, and it often is necessary to measure them using either an artificial head (Burkhard & Sachs, 1975) or miniature microphones inserted into the ear canals of a human subject (Wightman & Kistler, 2005).

Due to the combined acoustic effects of head and ears, ILDs show a complex dependency on frequency. They are around zero for sound sources in the median plane of the head and increase when sources are moved to one side; they also increase as a function of frequency, reaching values up to 20 dB at 4 kHz or higher (e.g., Fig. 2 in Bronkhorst & Plomp, 1988, which presents artificial-head data for a single source in an anechoic environment). When target and interfering sounds originate from different locations, their ILDs will normally be different, resulting in an SNR that is, on average, higher at one ear than at the other. The simplest way to predict the effects of ILDs on speech intelligibility is to equate binaural performance with that for the ear with the highest average SNR. A somewhat better method is to determine the ear with the highest SNR per frequency band and to combine these “best bands” in the calculation of speech intelligibility. The most sophisticated method, not used in current models, would be to perform a spectrotemporal analysis of the SNRs at both ears and calculate binary masks, indicating which ear is “better” in each time-frequency cell. Brungart and Iyer (2012) showed that a monotic signal created by applying such masks to the left and right signals and summating the results is equally intelligible as the original binaural stimulus, which suggests that the auditory system is indeed integrating “glimpses” of speech information fluctuating rapidly across ears and across frequency.

While the dependence of ITDs and interaural decorrelation on acoustics and on frequency is actually less complex than that of ILDs, predicting their effects is not as straightforward. Fortunately, quantitative models of binaural signal detection have been developed that also can be applied to speech perception (Durlach, 1972; Colburn, 1973). The Equalization-Cancellation (EC) model developed by Durlach (1972) is currently most widely used. It assumes that the auditory system optimizes SNRs in separate frequency bands by combining left- and right-ear signals in such a way that the energy of interfering sounds is minimized. This optimization takes place in three steps: the levels of the two monaural signals are equated, their phases are shifted, and one signal is subtracted from the other. The cross-correlation function of the left and right interferer signals provides important input for the EC model, because the position of the maximum determines the phase shift that should be applied, and the maximum value (the interaural coherence) indicates how effective the cancellation will be. The model also assumes that auditory signal processing is hampered by internal noise, so that perfect cancellation will never happen. The internal noise is modeled by applying time and amplitude jitters to the left- and right-ear signals before the EC operation.

The models of binaural speech perception developed by Wan, Durlach, and Colburn (2010), Beutelmann, Brand, and Kollmeier (2010), and Lavandier et al. (2012) all combine the EC model with a “best band” prediction of effects of ILDs. They therefore yield comparable predictions while using somewhat different implementations. The approach of Lavandier et al. (2012) is interesting, because it uses an analytical expression to calculate directly the binaural unmasking in dB, which makes it computationally efficient. The implementation of Beutelmann et al. (2010) is more complex but has as advantage that it performs calculations in short timeframes, which means that effects of interferer modulations can be predicted as well.

Van Wijngaarden and Drullman (2008) developed a binaural version of the STI model, discussed above, that does not use the EC model but quantifies how modulations of the input signal are preserved in the interaural cross correlation function. Because this function depends on interaural delay as well as on time, the delay is chosen at which modulations are optimally preserved, i.e., at which the MTF is maximal. These binaural MTFs are calculated within nonoverlapping frequency bands^3^ and compared to the left- and right-ear monaural MTFs. Per band, only the largest of the three MTFs is used for the final STI calculation. This method is attractive, because it uses a relatively simple way to calculate unmasking, while remaining consistent with the existing STI standard (IEC, 2003). It, furthermore, is the only binaural model that predicts how the intelligibility of target speech deteriorates as a result of reverberation. However, it is not able to model effects of interferer modulations.

Another approach is taken in the descriptive model first proposed by Bronkhorst (2000) and later refined by Jones and Litovsky (2011). This model considers conditions where all sound sources have the same average level and where the target speech always comes from the front. It predicts the decrease of the SRT that occurs when one or more interferers are moved from the front to positions around the listener. It consists of two additive terms: one related to how close or spatially separated the interferers are, and the other to the symmetry of their configuration. Jones and Litovsky (2011) applied the model to data from five studies with up to three interferers and found very high correlations between measurements and predictions (ρ ≥ 0.93). This model, therefore, seems very useful in cases when one wants quick estimates of unmasking occurring in a variety of spatial configurations.

### Summary of Research into Masking and Unmasking

In actual “cocktail-party” conditions, peripheral masking and binaural unmasking inevitably affect speech perception. Quantifying how much (un)masking occurs is, however, not easy, because it depends on a multitude of factors related to the speech signals, the environment, and the listener. Fortunately, powerful psychoacoustic models have been developed in the past decades that can deal with all of these factors and are able to generate sufficiently accurate predictions, using only a limited number of free parameters. Crucial properties of the models are (1) how speech information and its sensitivity to interfering sound and reverberation are quantified, and (2) which increase in speech information occurs during binaural listening.

The sEPSN model developed by Jørgensen et al. (2013) currently seems the most powerful approach to quantifying speech information, because it can deal with many factors, including reverberation, and requires only few parameters. Binaural listening is associated with three types of interaural differences: ILDs, ITDs, and interaural decorrelation. Given that ILDs cause differences in SNR between the ears, their effects can actually be predicted relatively easily using “monaural” speech perception models. Unmasking resulting from ITDs and interaural decorrelation can be adequately predicted by Durlach’s (1972) EC model or Van Wijngaarden and Drullman’s (2008) binaural STI. Although no single binaural model is available that addresses all relevant factors, related to source, environment, and listener, such a model actually can be developed relatively easily, because it is already known how any missing factor can best be quantified.

## Grouping of speech sounds

When extracting target speech from a multi-talker mixture, two different tasks need to be performed. One is to separate, at any time, target elements from other speech (*segregation*). The other is to connect elements across time (*streaming*). Bregman (1990) refers to these as simultaneous and sequential organization, respectively. That there can be substantial differences between these tasks is illustrated by results obtained with the Coordinate Response Measure (CRM) task, a speech intelligibility task used extensively in studies of informational masking (Bolia, Nelson, Ericson & Simpson, 2000). This task uses phrases of the form “Ready < call sign > go to < color > <number > now.” There are 8 call signs (e.g., “Baron”), 4 colors and 8 numbers, resulting in 256 possible phrases, spoken by 4 male and 4 female talkers. Listeners are asked to only attend to a phrase containing a specific call sign and to respond both the color and the number of that phrase. Multi-talker conditions are created by presenting phrases with different call signs, numbers, and colors at the same time. When same-sex talkers are used in such conditions, scores can be relatively poor (approximately 60 % when target and interfering speech have the same level), but almost all errors are colors and/or numbers of the nontarget sentence (Brungart, 2001). This means that listeners have little trouble segregating the two phrases, but they find it difficult to group the words in the correct stream.

Another distinction introduced by Bregman (1990) is that between “primitive” and “schema-based” grouping. Primitive grouping is supposed to take place preattentively, acting in a ”symmetric” way. It attempts to disentangle all superimposed sounds so that they are accessible for further processing, without “favoring” or selecting one specific sound. Schema-based grouping, on the other hand, is thought to rely on learned and/or effortful processes that make use of specific stored sound patterns. It also is thought to create a “figure-ground” distinction, which implies that it selects target information from other input, just as attention does. Bregman (1990), however, does not link attention directly to schema-based grouping. He indicates that such grouping could also take place preattentively, as long as it is based on learned schemata. Although the concept of primitive grouping seems useful, because it is linked to basic acoustic features that have been studied extensively, schema-based grouping is a more problematic notion, given that it is not easy to separate attentive from preattentive processing, and, especially in the case of speech, it is challenging to differentiate learned from innate schemata (e.g., Goldstone and Hendrickson, 2009). Furthermore, it is difficult to study because of the multitude of possible schemata and types of learned behavior that can be involved.

Given that several reviews of research on auditory grouping are available (Bregman, 1990; Darwin & Carlyon, 1995; Darwin, 1997; Darwin, 2008; Moore & Gockel, 2012), this overview focuses on research with speech that includes conditions in which target and interfering stimuli are presented simultaneously. First, two types of (“primitive”) grouping cues will be considered that dominate the recent literature: those based on voice characteristics and those related to spatial separation of target and interfering sources. Other cues, e.g., those based on language, are discussed in the third subsection.

### Grouping Based on Voice Characteristics

As already noted by Bronkhorst (2000), speech intelligibility is generally better when target and interfering speech are uttered by different-sex instead of same-sex talkers. Brungart (2001), for example, found that performance for the CRM task at negative SNRs differs approximately 20 percentage points between these talker combinations. An even larger difference—approximately 40 percentage points—occurs when the same voice is used as target and interferer. Festen and Plomp (1990) also compared different-sex with same-talker interference using a sentence intelligibility task and observed an SRT difference of no less than 6–10 dB.

Darwin, Brungart, and Simpson (2003) have looked more closely at the voice characteristics associated with differences in talker gender. They considered how fundamental frequency (F0) and vocal tract length affect CRM task performance. These parameters can adequately model the difference between male and female speech in synthesized speech (Atal & Hanauer, 1971). Maximum F0 changes and vocal tract length ratios used in the study were 1 octave and 1.34, respectively, which cover the differences between natural male and female speech (Peterson & Barney, 1952). It appears that CRM scores increase monotonically as a function of both parameters, but that the increase is somewhat higher for F0 changes than for vocal tract length changes. Interestingly, the effect of using an actual different–sex talker is larger than the sum of *individual* effects of F0 and vocal tract length, but around the same as the *combined* effect. In other words, changes of F0 and vocal tract length have, together, a superadditive influence on speech intelligibility. Note that Darwin et al. (2003) used natural fluctuations of the pitch contours of target and interfering speech, which were kept intact when the speech was resynthesized. Somewhat different effects of F0—a larger increase of scores for small differences and a dip occurring at one octave—were found in earlier studies that used completely monotonous speech (Brokx & Nooteboom, 1982; Bird & Darwin, 1998), probably due to partial fusion of target and interfering sounds.

Given that F0 is such a strong grouping cue, it is somewhat surprising that differences in F0 contour have much less effect. Binns and Culling (2007) used target and interfering speech with normal, flat (monotonous), and inverted F0 contours and found an increase of the SRT of up to 4 dB when the F0 contour of the target speech was manipulated but no significant effect of manipulations of the interfering speech. Thus, whereas F0 contour appears to be important for intelligibility (as shown previously by Laures & Weismer, 1999), differences in F0 contour between target and interfering speech do not seem to improve segregation of concurrent speech signals.

The results of Brungart (2001) mentioned above already indicated that voice characteristics that allow simultaneous grouping do not necessarily provide enough information for sequential grouping. In line with this, Brungart, Simpson, Ericson, and Scott (2001) showed that providing a priori information about the target talker by using the same talker in a block of trials helped to prevent different-sex, but not same-sex confusions (errors where the reported color and number in the CRM task were uttered by a different-sex or same-sex interferer, respectively). Apparently, such a priori information only cues the sex of the target talker.^4^ Furthermore, the modality used to present the information does not seem to matter. As shown by Helfer and Freyman (2009), who used a speech perception task similar to the CRM task, results did not depend on whether the target talker could be identified using a key word presented visually, or using an auditory “preview” of the target talker’s voice. Interestingly, Johnsrude et al. (2013) recently showed that listeners are much better at suppressing same-sex confusions when the target or interfering talker is highly familiar (the listener’s spouse).

### Grouping based on spatial cues

In studying spatial grouping cues, the most direct approach is to simply present target and interfering speech stimuli from different spatial locations. Such experiments show that a relatively small spatial separation can already lead to efficient segregation. Brungart and Simpson (2007), for example, found that a separation of only 10° of two voices is already sufficient to maximize performance on the CRM task. However, because spatial separation is normally accompanied by changes in audibility, the true contribution of grouping cannot be determined in this way. One solution for this problem is to minimize masking by making sure that the frequency spectra of target and interfering hardly overlap. Arbogast et al. (2002) realized this with sine-wave speech, created by filtering CRM sentences in multiple frequency bands, using the envelopes to modulate pure tones at the center frequencies of these bands, and subsequently summing together separate subsets of these tones to generate target and interfering signals. Such speech is perfectly intelligible after some training. Using a similar procedure, unintelligible sine-wave “noise” was generated with a frequency spectrum identical to that of the sine-wave speech. When target and interfering sounds were both presented from the front, the “noise” had much less effect on intelligibility than the speech interference did. This reflects the difficulty of grouping CRM phrases demonstrated earlier by Brungart (2001). The interesting finding was that this difference was reduced drastically (from more than 20 dB to approximately 7 dB in terms of SRTs) when a spatial separation of 90° was introduced. Thus, spatial separation can strongly reduce the influence of interfering speech even when audibility hardly changes.

Another way to separate audibility changes from effects of grouping was devised by Freyman and colleagues. They compared a baseline condition in which target and interfering speech came from the front (labeled F-F) with a special condition in which the interfering sound was presented both from the right side, at an angle of 60° and, slightly delayed, from the front (the F-RF condition). Thus, they made use of the precedence effect (the dominance of the first-arriving sound in localization) to create a *perceived* spatial separation without reducing energetic masking. Freyman et al. (2001) showed that this change of perceived location caused a gain of approximately 8 dB for interfering speech (at 60 % intelligibility) but of only 1 dB for interfering noise. Interestingly, Freyman, Helfer, McCall, and Clifton (1999) also used an F-FR condition in which the two interfering signals were reversed; i.e., the delayed copy was presented from the spatially separated source. This generated a much smaller shift in perceived location but about the same gain with respect to the FF condition. Spatial separation, thus, appears to be very effective in improving segregation of speech sounds.

Because spatial segregation introduces both ILD and ITD, it is of interest to look at the individual contributions of these cues. The general approach to studying this has been to present signals with one of these cues through headphones and then measure how segregation diminishes when other grouping cues are added. The “shadowing” studies conducted by Cherry (1953) and others are, in fact, early examples because they combined “infinite” ILD with various manipulations of target and/or interfering speech.^5^ Treisman (1960), for example, asked listeners to shadow speech presented to one ear while sudden switches of target and nontarget speech were introduced. Listeners generally maintained their shadowing performance and only occasionally reproduced words from the nontarget ear after a switch. This demonstrates that grouping based on ILD is dominant but nevertheless can be superseded by grouping based on speech properties (i.e., voice characteristics as well as contextual cues). Cutting (1976) used a different paradigm in which 2-formant synthetic syllables were presented dichotically, and performance was determined for complementary signals (e.g., different formants) or conflicting signals (e.g., pairs of formants differing in their initial parts). Note that the common F0 and the temporal alignment of the left and right signals acted as strong grouping cues in these conditions. His results show perfect integration for complementary stimuli and 50–70 % integration for conflicting stimuli. Although this indicates that grouping is affected, it does not mean that the two sounds are always fused. In the study of Broadbent and Ladefoget (1957), who used similar (complementary) stimuli, only about half of their listeners indicated that they heard just one sound. Darwin and Hukin (2004) later showed that fusion, in fact, depends on the presence of overlapping spectral information at the left and right ears. When sharp filters without overlap are applied, listeners always report hearing two sounds.

Interestingly, ILD-based grouping can be much less robust when there are multiple interfering sounds. Brungart and Simpson (2002) found that when one target and one interfering talker are presented to one ear, performance decreases considerably (by up to 40 percentage points) when a second interfering talker is presented to the contralateral ear. Even more surprising is their finding that this decrease stays almost the same when the contralateral interfering speech is strongly attenuated (by up to 15 dB). Further research by Iyer, Brungart, and Simpson (2010) revealed that this “multi-talker penalty” only occurs under specific conditions: at least one of the interfering signals should be similar to the target signal (so that they are easily confused) and the overall SNR should be below 0 dB.

While it appears to be difficult to disrupt segregation based on (large) ILDs, several studies indicate that ITD-based segregation is less robust. Culling and Summerfield (1995) showed that artificial vowels consisting of two narrow noise bands can be easily segregated when they differ in ILD but not when they differ in ITD. Using a different paradigm that looked at the degree to which a single harmonic was integrated in the percept of a vowel, Hukin and Darwin (1995) also found that ITD causes much weaker segregation than ILD. These findings are remarkable, because ITD is known to be a potent spatial cue for sound localization (Wightman & Kistler, 1992) and speech perception in noise (Bronkhorst & Plomp, 1988). Results of other studies are, however, much less clear-cut. Drennan, Gatehouse, and Lever (2003), for example, replicated the Culling and Summerfield experiments using natural ITDs and ILDs, derived from artificial-head recordings. They not only found that the majority of their listeners could now segregate the noise stimuli using ITD, but also that performance for ILD was only slightly better than that for ITD. An important factor appeared to be the inclusion of onset ITDs. Were these absent, so that only ongoing ITDs remained, performance decreased. Another study comparing ITD- and ILD-based segregation was conducted by Gallun et al. (2005). They used the sine-wave speech stimuli devised by Arbogast et al. (2002) and looked at performance differences occurring between a monotic baseline condition, in which target and interferer were presented to one ear and dichotic conditions where the ILD or ITD of the interferer was varied. They, in essence, found that any interaural difference that generated a perceived lateralization shift also allowed segregation of target and interfering speech.

Further work on ITD-based grouping was conducted by Darwin and Hukin (1999), who studied segregation of words embedded in sentences. In their paradigm, listeners focused on one of two sentences presented simultaneously but with different ITDs, and their task was to select a target word within that sentence that coincided with a competing word in the other sentence. Care was taken to ensure that these words could not be identified using other (semantic, prosodic, or coarticulatory) cues. Performance not only was high (>90 % for ITDs larger than 90 μs) but also did not change when F0 difference was pitted against ITD (i.e., sentences had different ITDs and F0s but the target word had the same F0 as the competing sentence). A follow-up study by Darwin and Hukin (2000) that used synthesized sentences with natural prosody showed that grouping based on ITD will only break down when prosody, F0 difference, and a (large) vocal-tract difference are all working against it. ITD, thus, appears to be a quite strong cue for segregation, provided that streaming can build up and natural onset cues are included in the stimuli.

In real-life conditions, cues are mostly working together and not against each other, and it is of interest to determine how effectively cues are combined. Culling, Hawley, and Litovsky (2004) measured intelligibility for target speech presented from the front and three-talker interference presented from various combinations of source positions and processed their stimuli such that they contained only ITD or ILD, or both ITD and ILD. They found that recognition performance was always better for the combination than for individual cues, but that the improvement was sub-additive. A different cue combination-frequency and spatial location—was studied by Du et al. (2011). They used synthetic vowels with either the same F0 or a small F0 difference that were presented from the front or from locations at ±45°. Additive effects of the two cues were found both in the behavioral scores and in MEG responses measured simultaneously. The latter results are consistent with earlier electrophysiological studies showing additivity of responses to combinations of frequency and location (Schröger, 1995) and frequency and intensity (Paavilainen et al. 2001). Such additivity not only indicates that the auditory system can integrate these cues effectively but also suggests that they are processed in separate brain regions (McLachlan & Wilson, 2010).

### Grouping based on other cues

Several studies mentioned above have used low-level grouping cues, such as harmonicity and temporal alignment of speech items to counteract segregation introduced by spatial cues. Although these cues are clearly potent, I will not consider them further, because they are less representative of real-life listening conditions and because in-depth reviews of this work are already available (Darwin, 2008, Moore & Gockel, 2012). Many other cues are present in speech that probably also affect grouping. Examples are speaking style (e.g., timing, stress pattern), timbre, linguistic variability (e.g., native vs. nonnative speech), and various types of contextual (e.g., semantic, syntactic, and coarticulatory) information. While a lot is known about their effects on intelligibility, as discussed in the section “How Special is Speech,” we know surprisingly little about how they affect grouping. This is probably, because specific measures were taken in most studies to remove or control these cues to make effects of other cues more salient. Freyman and collegues, for example, used meaningless sentences with target words at fixed locations, which provide no semantic or syntactic information. Also in the popular CRM task, listeners cannot benefit from such information, nor from differences in timing or stress pattern, because all phrases have the same structure and target words are drawn randomly from fixed sets. Nevertheless, some relevant results are available that were obtained by manipulating these cues in the interfering instead of the target speech.

**Fig. 1:**
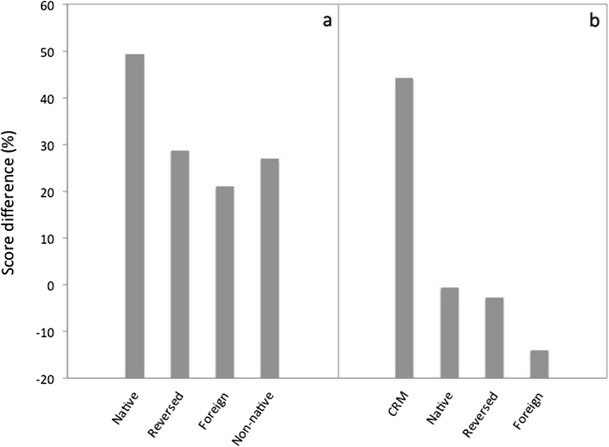
Measures of “informational masking” derived from data collected by Freyman et al. (2001; panel a) and Iyer et al. (2010; panel b) in conditions where target speech was presented together different types of two-talker interference. Shown are differences between speech perception scores, averaged over SNRs of −8, −4, and 0 dB, for reference and test conditions. **a** Scores for test conditions using F-RF presentation from which scores for reference conditions, using FF presentation of the same sounds, were subtracted. **b** Data obtained by subtracting scores for test conditions with speech interference from a reference condition with two modulated noise signals.

Freyman et al. (2001) performed experiments where the interfering speech was spoken by native or nonnative talkers, time-reversed, or spoken in a foreign language, unknown to the listeners. The precedence-effect-based paradigm discussed above was used; all talkers were female. For all types of interference, a clear difference was found between results for the F-RF and F-F conditions, demonstrating that grouping was always facilitated by the introduction of a (perceived) spatial separation (Fig. 1a). The difference was, however, much larger for the forward native speech than for the other types of speech. This indicates that grouping of multiple speech sounds is affected by any type of interfering speech, irrespective of its intelligibility, but suffers in particular from normal speech uttered by native talkers. Iyer et al. (2010) performed somewhat similar experiments with the CRM task, using not only interfering CRM phrases but also other native, foreign or time-reversed interfering speech. Their results are summarized in Fig. 1b. As expected, performance was worst for the CRM interferers, which have the same structure and timing as the target phrases and thus maximize confusions. Performance was intermediate for interfering normal or time-reversed English speech and relatively good for foreign speech. Although these results are largely consistent with those of Freyman et al. (2001), it is surprising that no difference occurred between normal and time-reversed speech. Perhaps this is an artifact caused by the use of CRM sentences, which could induce listeners to just focus on target words and dismiss semantic information altogether.

### Summary of Research into Grouping

The difficult task of a listener to disentangle target from interfering speech is facilitated by the presence of different types of grouping cues. Research shows that voice characteristics contain effective cues and that in particular female and male voices are easily segregated. It can be surprisingly difficult to track voice characteristics over time, which means that perfect segregation is not always accompanied by perfect streaming. Study of individual cues reveals that F0 and vocal tract differences both contribute to grouping and that they have a superadditive effect when combined. Differences in intonation (F0 contour), however, do not seem to act as grouping cue. Other important cues are the ILDs and ITDs occurring when different talkers occupy different positions in space. A large ILD acts as an extremely strong grouping cue, which can only be made ineffective by pitting a combination of other grouping cues against it. While ITD was found to be ineffective for segregation of certain artificial stimuli, it is around as effective as ILD when more natural stimuli are used. Given that the combination of ILD and ITD has an even stronger effect than each of the individual cues, it is not surprising that optimal segregation can already occur for a small spatial separation. When spatial separation is combined with other cues such as F0, additive effects on performance are found, suggesting independent processing of these cues by the auditory system.

Remarkably little is known about other grouping cues, related to e.g. language, pronunciation and redundancy. Studies in which the interfering speech was manipulated show that segregation is easier for nonnative or foreign, than for native interfering speech. Segregation is poorest when target and interfering speech have the same syntactic structure. This indicates that linguistic and syntactic differences facilitate grouping. Comparisons of the effects of normal and reversed interfering speech yield unequivocal results, which means that it is not yet clear whether semantic differences support grouping as well.

## Role of attention in selecting speech

Attention is currently mostly defined from an information-processing point of view, stressing the need for selection of information by a system that is capacity limited and largely serial in its central processing stages and in response selection and execution (Pashler, 1998). Attention is steered both by bottom-up sensory information (e.g., attentional capture by a novel stimulus) and by top-down processes (e.g., endogenous focusing on a specific spatial location). This implies that it is difficult to control attention experimentally, not only because one never knows whether subjects follow instructions telling them to focus their attention in a certain way but also because it is hard to prevent bottom-up capture. Shifts of attention also may be difficult to detect, because they can occur quite rapidly: within 100–200 ms (Spence & Driver, 1997).

Cherry’s (1953) interest in the cocktail-party problem resulted in a novel paradigm, in which listeners are asked to shadow speech presented to one ear while ignoring signals presented to the other ear. This paradigm is important, because it provided most, if not all material on which the early theories of attention were based. These theories all state that attention operates as a filter that selects part of the incoming auditory information, but they differ in their predictions of which unattended information is processed. While the “early selection” theory states that only low-level signal features are processed preattentively (Broadbent, 1958), the “late selection” theory claims that all input is processed up to a semantic level (Deutsch & Deutsch, 1963). An intermediate view is provided by the “attenuation” theory of Treisman (1964), which proposes that unattended information is indeed processed up to a high (semantic) level, but with reduced resources and thus more slowly. Although some clever experiments were performed in the attempts to prove one or the other theory (Moray, 1959, Treisman, 1960), it appears that this research, and the modeling based on it, suffers from two basic flaws. One flaw is that possible shifts in attention to the nontarget ear were at best poorly controlled, and its possible effects were not monitored (Holender, 1986). The other flaw is that, while the use of the term “filter” implies that sensitivity is measured as a function of a certain independent variable, this variable is not made explicit, nor manipulated.

### Is Attention Required for Speech Processing?

In order to shed more light on the results of the classic shadowing studies, Cowan, Wood, and colleagues replicated several experiments, analyzing in detail the shadowing output and its timing (Cowan & Wood, 1997). When they repeated Moray’s (1959) classic experiment, in which the listener’s own name was unexpectedly presented to the nontarget ear, Wood and Cowan (1995a) reproduced the finding that about one third of the listeners recalled hearing their name afterwards, but also showed that the shadowing errors and/or response lags of these listeners increased significantly in the seconds following the occurrence of their name. No decrease in performance was found for listeners who did not notice their name or were presented with other names. Similar results were observed for listeners presented with a fragment of time-reversed speech embedded in normal speech (Wood & Cowan, 1995b). While this indicates that attention switches do occur and that attentional resources are required for consolidation of information in long-term memory (LTM), it also suggests that some preattentive semantic processing of speech must be going on to trigger the switches. Evidence for such preattentive processing also was provided by Rivenez, Darwin, and Guillaume (2006), who studied dichotic priming with a paradigm that combines high presentation rates (2 words/s) with the addition of a secondary task to discourage attention switches to the nontarget ear. Despite these measures, they found a clear priming effect: response times to a target word were lowered when the same word was presented directly before it to the nontarget ear.

Even more compelling evidence for preattentive processing emerges from recent mismatch-negativity (MMN) studies. The MMN is an event-related potential (ERP) component that occurs when infrequent “deviant” stimuli are inserted in a series of “standard” stimuli (Näätänen, Gaillard, & Mäntysalo, 1978). It is resistant to influences of attention and is therefore thought to reflect a preattentive process that compares the current auditory input with a memory trace that encodes the regularity of previous input. In their review of MMN work on language processing, Pulvermüller and Shtyrov (2006) conclude that the MMN is sensitive to different types of manipulations, representing separate levels of language processing. MMNs are for example found in response to (1) pseudowords versus words (lexical level), (2) action words versus abstract words (semantic level), and (3) words in grammatically correct sentences versus words in ungrammatical strings (syntactic level). Because MMN data also depend on physical stimulus differences, Pulvermüller and Shtyrov (2006) only consider studies where such effects are prevented. However, as these authors note themselves, another critical aspect of most MMN work involving speech stimuli is that attention is only loosely controlled, so that it is not clear to what degree results are modulated by attention. An exception is a study by Pulvermüller, Shtyrov, Hasting, and Carlyon (2008), who studied MMN responses to syntactic violations in speech presented to one ear, while subjects had to detect oddball stimuli among standard tones presented to the other ear (and watched a silent video as well). In the control condition, they were presented with the same stimuli but did not perform the (attentionally demanding) task. The results show that the early part of the MMN (<150 ms) is immune to the manipulation of attention, which indicates that there is indeed preattentive processing of speech up to a syntactic level.

### Attending to a target voice

What are the sound features that help us attending to a target voice? This question was already answered partly in the previous sections, because any stimulus that can be recognized in behavioral speech segregation experiments first must have been selected. This means that voice differences and spatial cues also are effective cues for selection. However, we can only learn more about selection itself when paradigms are used in which attention is manipulated. Implicit manipulation took place in a number of studies that were already discussed. Brungart et al. (2001) included conditions where the target voice was either varied across trials or kept constant, so that listeners either had to refocus attention on (the voice of) each individual call sign or could maintain attention on the same voice all the time. The manipulation just suppressed different-sex confusions, which indicates that sustained attention can only be focused on relatively coarse voice characteristics. In another study based on the CRM task, Kidd, Arbogast, Mason, and Gallun (2005) manipulated information about the target sentence and the target location. They used a paradigm in which three phrases (spoken by make talkers) were presented simultaneously from equidistant sources on a 120°-arc in front of the listeners. In a block, the call sign was cued either before or after presentation of the phrases, and the probability of the target location had a constant value between 0.33 (chance) and 1.0. It appeared that knowing the location yielded maximal performance, also when the call sign was provided afterwards, but that knowing the call sign while being uncertain of the location caused a reduction of 10–40 percentage points. These results confirm the difficulty listeners have in keeping track of a target voice, and also demonstrate that spatial location provides relatively strong cues.

Several studies on spatial auditory attention have used nonspeech (and nonsimultaneous) stimuli but are nevertheless interesting, because they combine behavioral and electrophysiological measures. Teder-Sälejärvi and Hillyard (1998), for example, used a spatial attention task in which noise bursts were presented from 7 regularly spaced loudspeakers spanning a 54°-arc in front of the listeners. Standard stimuli as well as (infrequent) target stimuli with a different frequency bandwidth were generated by all loudspeakers, but the listeners were required to respond only to targets delivered by one specific loudspeaker. Behavioral error rates were low, indicating that attention had a relatively narrow spatial focus (within ±9°, which is consistent with the spatial resolution found in the abovementioned study of Brungart and Simpson, 2007). Interestingly, ERPs recorded simultaneously revealed a similar spatial tuning at latencies around 300 ms poststimulus but a broader tuning for earlier latencies. This suggests that the spatial tuning of attention operates in different stages with increasing sharpness. Similar results were obtained by Teder-Sälejärvi, Hillyard, Röder, and Neville (1999), who also found that spatial tuning for a source at a horizontal angle of 90° was around twice as broad as for a location in front. This is consistent with the fact that sound localization accuracy decreases for more lateral source positions (Middlebrooks & Green, 1991).

Hink and Hillyard (1976) developed a paradigm that enabled them to measure ERPs using speech stimuli. They asked listeners to attend to one of two stories presented simultaneously to the left and right ears and used synthesized phonemes, mixed with the stories, as probe stimuli. Although ERPs were smaller than when the probes were presented alone, a clear effect of attention was observed: N_1_ responses were significantly higher for probes coming from the target side. Recently, Lambrecht, Spring, and Münte (2011) used the same paradigm for stimuli presented in a (simulated) free field. Listeners had to attend one of two concurrent stories emanating from sources at horizontal angles of ±15°, and ERPs were elicited by probe syllables cut out from the stories. The probes were presented either from the same locations as the stories or from locations at more lateral angles (±30° or ±45°). An increased negativity was found for probes coming from the target location compared with those coming from the mirrored location, but latencies were higher (>300 ms) than those observed by Hink and Hillyard (1976). The results are consistent with those of other studies using similar paradigms, which also revealed late attention-related responses for dichotic (Power, Foxe, Forde, Reilly, & Lalor, 2012) and free-field (Nager, Dethlefsen, & Münte, 2008) speech presentation. The deviant results of Hink and Hillyard (1976) might be caused by their use of synthetic probe stimuli that do not require speech-specific processing and can draw attention based on simple acoustic differences. Interestingly, Lambrecht et al. (2011) found that probes coming from more lateral positions yielded (strongly) increased negativity, but only for the 45°-angle at the target side. The authors attributed this to a reorienting response, indicating that attention was relocated to the original task after momentary distraction by the probe.

### Exogenous attention to speech

The results of Lambrecht et al. (2011) and also the classic finding that occurrence of one’s own name in nontarget sounds affects performance on a primary task (Moray, 1959; Wood & Cowan, 1995a) are examples of findings that can be explained by exogenous shifts of attention. Another example is the irrelevant sound effect—the finding that performance on (visual) memory tasks is impaired when task-irrelevant sounds are presented during encoding or retention of to-be remembered items (Colle & Welsh, 1976; Neath, 2000). It has been proposed that this deficit is primarily due to involuntary shifts of attention away from the primary task (Cowan, 1999; Bell, Röer, Dentale, & Buchner, 2012), although there also are alternative explanations, suggesting a direct interference with memory processes (Jones, 1993). Given that the effect mainly concerns the functioning of (visual) memory, this research will not be discussed further in this review. Other research into exogenous auditory attention has borrowed tasks from visual research but has used nonspeech stimuli. Spence and Driver (1994), for example, developed an auditory version of the classic spatial cueing task (Posner & Cohen, 1984) and showed that presenting an auditory cue just before a target sound reduced response times when the cue came from the target, instead of the mirrored side. Dalton and Lavie (2004) developed a paradigm based on Theeuwes’s (1992) attentional capture task, in which a target tone with deviating frequency (or level) had to be detected in a tone sequence that also could contain an irrelevant tone with deviating level (or frequency). They found that the irrelevant singletons impaired performance, indicating that they indeed captured attention. In a recent study by Reich et al. (2013), both nonspeech and speech stimuli were used in a paradigm where participants responded to the second syllable of a spondee or the duration of a tone, while irrelevant deviations were introduced by changing the first syllable of the spondee or its pitch, or the frequency of the tone. All deviations increased behavioral error rates and response times, and elicited P3a components in the ERPs, consistent with the occurrence of involuntary shifts of attention.

More insight into the relationship between speech processing and exogenous attention is provided by a series of experiments conducted by Parmentier and colleagues (Parmentier, 2008, 2013; Parmentier, Elford, Escera, Andrés, & San Miguel, 2008; Parmentier, Turner, & Perez, 2014). These researchers used different cross-modal tasks to measure effects of irrelevant auditory stimuli on visual identification tasks. The paradigms are based on earlier studies employing non-speech stimuli (Escara, Alho, Winkler, & Näätänen, 1998). Participants were asked to classify digits or identify the direction of an arrow. These visual stimuli were preceded by standard auditory stimuli (mostly tones) or by infrequent deviant stimuli that could be noise bursts, environmental sounds, or utterances that were congruent or incongruent with the visual task (the words “left” or “right”). Stimulus-onset asynchronies (SOAs) varied between 100 and 350 ms. Several interesting results were found. The basic finding, replicated in all studies, is that deviant irrelevant stimuli reduce performance, as reflected by increased response times and decreased accuracy. As shown in the first study (Parmentier et al., 2008), this reduction is unaffected by visual task difficulty but it disappears when an irrelevant visual stimulus is presented just after the deviant auditory stimulus at the location of the subsequent target. This suggests that attention is indeed captured by the deviant stimulus, but can be refocused quickly on the target position. Follow-up experiments (Parmentier, 2008) not only revealed that incongruent deviants (e.g. the word “left” presented before an arrow pointing to the right) disrupt performance more than congruent deviants, but also that the difference between the two (semantic effect) is independent of whether standards are acoustically similar to or different from the deviants (novelty effect). As shown by Parmentier et al. (2014), this semantic effect decreases substantially when the visual target has varying SOA and 50 % probability of occurrence, indicating that the degree to which the deviants are semantically processed depends on how reliably occurrence of the target is predicted by the auditory stimulus. Taken together, the results indicate that deviant auditory stimuli incur exogenous shifts of attention but that further processing of these stimuli depends on their relevance for the task at hand, and, thus, on endogenous factors such as task goal and listening strategy.

### Attending to Changes in Talker Voice and Location

In real-life listening we often switch our attention between sounds and/or source locations. Several studies have investigated this by introducing target uncertainty, thus discouraging listeners to maintain focus on a single sound source. In the study by Kidd et al. (2005), discussed above, listeners performed a CRM task while uncertainty in target identity (knowledge of the call sign) and/or location was manipulated. Ericson, Brungart, and Simpson (2004) performed a similar study in which listeners always knew the call sign but where uncertainty in the target voice and/or location was introduced. These studies in particular demonstrate how the different cues affect performance: knowing only target identity or target voice yields lower scores that knowing the location and knowing combinations of cues always improves scores. They, however, provide no information on actual switch costs, because it is unknown whether and how listeners refocus their attention in these conditions. A more direct study of the dynamics of attention was conducted by Koch, Lawo, Fels, and Vorländer (2011), who measured how switches of the target talker affected response times for an auditory classification task. They used dichotic speech stimuli, consisting of number words spoken by a female and a male talker, and provided listeners with a visual cue signaling the target talker’s gender. Using a paradigm that enabled them to separate visual from auditory switch costs, they found that the latter were around 100 ms, independent of cue SOA (which was either 100 or 1000 ms). However, because the target ear was randomized in the experiments, these costs actually represent a combination of switching between voices and (in 50 % of trials) switching between ears.

**Fig. 2:**
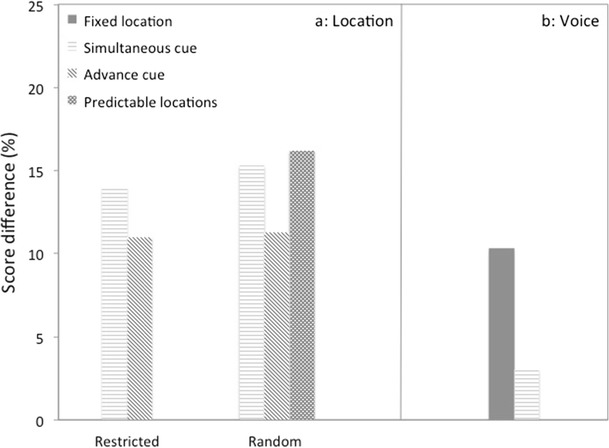
Data from experiments of Best et al. (2008) and Best et al. (2010), in which one target and four interfering strings of digits were presented from different loudspeakers placed in an arc in front of the listener. **a** Increases in scores occurring when target digits are presented from a fixed location, instead of from “restricted” locations changing at most one loudspeaker position at a time, or from random locations. The changes could be cued with lights either at the time of change or in advance. The condition “predictable locations” employed the same sequence of locations throughout a block. **b** Increases are shown occurring when the string of target digits was spoken by a single voice, instead of different voices for each digit. The “simultaneous cue” condition in this case used random locations. All results are for an inter-digit delay of 250 ms, except those for the “predictable locations” condition, to which a correction factor was applied because they were only measured for a delay of 0 ms

Best, Ozmeral, Kopčo, and Shinn-Cunningham (2008) and Best, Shinn-Cunningham, Ozmeral, and Kopčo (2010) conducted a series of experiments that provide the most detailed insight into effects of switches of target voice and location. They used 5 evenly spaced loudspeakers on a 60°-arc in front of the listeners. Target and interfering stimuli were sequences of four digits uttered by male talkers. The target, which was always presented together with four (different) interferers, could come from a fixed loudspeaker, or consecutively from different loudspeakers. Listeners had to repeat the 4 consecutive target digits in order. Surprisingly, the results showed that the reduction in scores resulting from location changes was resistant to all kinds of measures designed to aid refocusing of attention. These included cueing the location with lights, either at the moment of change or in advance, using only changes to the nearest loudspeakers, and using repeated sequences of locations (Fig. 2a). Another surprising finding was that the beneficial effect of keeping the target voice the same almost disappeared when the target location started shifting (Fig. 2b). Moving the target around thus disrupts the advantage of using voice identity as cue, which suggests that a position shift causes a sort of “reset” of the system.

Best et al. (2008, 2010) also provided insight in the time constants involved in tracking shifts in talker and location. They found that the cost of switching location decreased when the interstimulus delay was increased to 1000 ms, but not to zero. Furthermore, they observed that performance for a fixed target location improved over time, irrespective of any switches in talker identity. Even for the 1000-ms delay (sequence duration of approximately 5 s), the improvement was still considerable (approximately 10 percentage points). Brungart and Simpson (2007), who conducted an experiment with two to four talkers using sequences of CRM trials with varying spatial uncertainty, found a similar pattern of results that spanned even a much longer time period. The improvement they found as a result of keeping the target location fixed continued up to the 30th trial (a sequence duration of approximately 160 s). Note, however, that there was uncertainty about the location of the target talker in this experiment so that the slow improvement occurring when the target location remained the same for some time may also be due to the fact that the listeners gradually changed their listening strategy to one optimized to a nonmoving talker.

### Summary of research into attention

Whereas research into auditory attention stagnated for some time after the early shadowing studies approximately 50 years ago, an increasing number of studies is now being conducted, using various behavioral paradigms as well as electrophysiological measures. It appears that attention is very versatile. It is not just able to suppress speech presented to one ear when we are listening to the other ear—it also can focus on a relatively narrow spatial region around a target voice (within ±10°) or on characteristics of a target voice mixed with interfering speech. These features are not equally effective: listeners find it easier to sustain focus on a location than to keep track of a voice, even if a relatively short message is uttered.

The most surprising trick that attention can perform is that it can base selection not just on basic features, such as location, but also on sophisticated semantic cues, which are processed preattentively. Indications for such processing already emerged from many behavioral studies, but it was not until recently that convincing proof was provided by MMN measures. Such processing is particularly useful for exogenous triggering of attention: it enables us, for example, to pay attention to someone suddenly mentioning our name. We would expect that capacity limitations must restrict how much unattended speech can be processed, and recent experiments by Parmentier et al. (2014), indeed, provide evidence for this. The depth of processing seems to be related to the (task-)relevance of the sound.

The weak spot of auditory attention seems to be that it is remarkably sluggish. That sudden changes in talker identity are penalized is perhaps not too surprising, given that this is unusual and the system has to attune to subtle cues in individual voices. A more unexpected finding is that changes in talker location seem to “reset” this tuning process: speech perception is no better when one can follow a single talker successively moving to different positions in space, than when different voices are presented from each of these locations, so that changes in location are always coupled with changes in talker identity. Furthermore, optimal tuning to location and voice takes a pretty long time. Perhaps this is the price that has to be paid for superb selectivity.

## Conceptual Model of Early Speech Processing

This section describes a conceptual model of preattentive speech processing and auditory attention that intends to incorporate most results reviewed above. It builds upon earlier models, in particular on the model of conscious and unconscious auditory processing published recently by Näätänen et al. (2011) and to a lesser degree on the generic sensory-information-processing model of Cowan (1988). Because it is primarily intended as a high-level model integrating behavioral findings, it does not attempt to include elements of neurobiological models (such as that developed by McLachlan and Wilson, 2010). Also, because of the focus on early processing, it does not consider results of psycholinguistic research that addresses higher-level speech processing.

### Structure and Operation of the Model

**Fig. 3:**
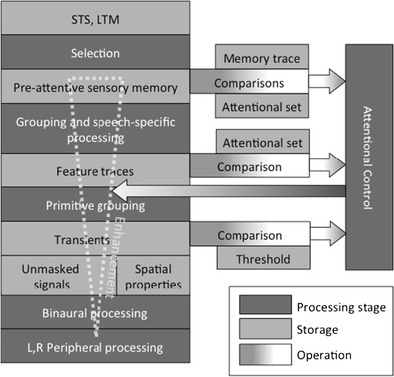
Conceptual model of early speech processing. After peripheral and binaural processing, transients can already trigger attention. Primitive grouping (e.g., based on spatial location or F0) represents a subsequent stage, allowing efficient selection. More sophisticated features, such as syntactic and semantic information, are processed at a higher level and enable selection based on complex information. An important element of the model is a feedback loop, initiated by attentional control, inducing enhancement of to-be-selected input. See the text for more details

As shown in Fig. 3, the model contains processing stages, storages, and operations linked to attention, which have been given different shadings. The processing stages are (1) peripheral and binaural processing, (2) primitive grouping, (3) further grouping and speech-specific processing (such as processing of lexical information), (4) selection, and (5) attentional control. There are storages for (a) unmasked signals and spatial properties, (b) transients, (c) primitive features, (d) preattentive sensory information, and (d) short-term and long-term information, including thresholds, sets, and traces used for triggering attention. The model assumes that processing is largely feedforward but also is influenced by a feedback loop, governed by attention, inducing selective enhancement of signals being processed. This is indicated in Fig. 3 by the dotted triangle and by the arrow linking it to attentional control. The enhancement operates on signals that have triggered attention, but extends to stages before the one at which the trigger occurred. Support for this comes from EEG and imaging studies showing effects of attention on neural processing in the nonprimary (Ahveninen, Hämäläinen, Jääskeläinen, Ahlfors, Huand et al., 2011) and primary (Woldorff et al., 1993) cortex, and from evidence of selective processing already taking place at the peripheral level. Scharf, Quigley, Peachey, and Reeves (1987; see also Scharf, 1998), for example, studied the effect of attention on detection of tones in noise and found that scores increased strongly when the tone frequency was close to or equal to an expected frequency. More recently, Allen, Alais, and Carlile (2009) showed that binaural unmasking also is influenced by attention: they found that the spatial release from masking of speech stimuli disappeared when the target speech was presented from an unexpected location. Given that attention controls enhancement of preattentive signals, the selection stage itself can be seen as a relatively simple stage that just passes the strongest of the competing input signals.^6^

According to the model, attention can be triggered at multiple levels: two early levels, enabling “fast” selection, and a late level, used for “slow” selection. In each case, the trigger comes from a comparison between incoming information and information present in memory. “Fast” bottom-up selection is based on basic signal properties such as sound level or fundamental frequency. Attention is drawn when a transient—for example a sudden loud sound—exceeds the corresponding threshold. “Fast” top-down selection is based on primitive features, such as sound level, interaural differences, F0, and spectral envelope. These are compared to an attentional set determined by the task and goals of the listener. When listening to a talker at a certain spatial location, this set would contain the corresponding ITDs and ILDs. Focusing on a female voice among male voices would require comparison with an F0 range and with templates of spectral envelopes. “Slow” bottom-up selection occurs when a more complex deviation from regularity than a transient is detected. This is represented in the model by the comparison between a stored memory trace and a novel trace in sensory memory. It also occurs in the classic case when a listener recognizes one’s own name in unattended speech. Such speech is not enhanced and is therefore processed more slowly and with more risk of decay. However, when the speech item reaches the preattentive sensory memory and is compared with a generic attentional set containing relevant speech items (such as one’s own name), attention can nevertheless be drawn. A “slow” top-down route is the comparison of complex speech items that have passed through grouping and speech-specific processing, with items in an attentional set. This route is, for example, followed when one is listening for the occurrence of a specific message in a mixture of voices.

The model assumes a relatively straightforward interaction between attention and grouping. It supposes that all grouping occurs at a pre-attentive level (i.e., before selection takes place), which means that it acts on all sensory input, without requiring conscious effort, and reuses Bregman’s (1990) concept of primitive grouping. In the primitive grouping stage, signals are organized along basic features that can subsequently be used for comparison with an attentional set. The following grouping stage can be based on more complex speech properties, because it is combined with higher-level (lexical, semantic, and syntactic) speech processing. Because the model supposes that selective enhancement is largest at stages following the one that triggered attention (as illustrated by the shape of the dotted triangle), there is more bias towards the enhanced signals in higher-level than in primitive grouping. This corresponds with Bregman’s (1990) proposition that primitive grouping operates similarly on all input, while later grouping differentiates “foreground” from “background.” The model, however, also deviates from Bregman’s (1990) views because it does not include effortful/conscious grouping. This was done not only because of lack of experimental evidence but also because the range of properties that could underlie such grouping is almost limitless.

The model includes peripheral and binaural processing as initial stages, which may be seen as placeholders for quantitative models such as those presented above. There are, however, some issues that prevent such a simple merge. One is that it is not certain to which degree the psychoacoustic models represent auditory processing in a neurophysiologically plausible way, at least not beyond the cochlear level (for which most use commonly accepted representations of critical-band filtering). A solution for this could emerge from recent research into the relationship between speech perception and neuronal oscillations (Zion Golumbic et al., 2013). This research focuses on the role of envelope fluctuations and thus might provide a basis for envelope-based models such as that of Jørgensen et al. (2013). Another issue is that the models require knowledge of the target speech signal—its level, frequency spectrum, and perhaps also modulation spectrum—which presents a logical problem in the current model because the target speech signal only emerges in the later grouping stages. There are possible solutions for this problem—all potential target signals may be processed in parallel, or the target speech may be “highlighted” by the selective enhancement initiated by attention, but this is all rather speculative. Clearly, further research is required to shed more light on these issues.

### Relationship with earlier models

As mentioned above, the model is inspired mainly by that developed by Näätänen et al. (2011), which is based on a large body of electrophysiological work, primarily on the MMN and the N1 (Näätänen & Picton, 1987). Many elements of that model were incorporated, but there are several significant discrepancies. An obvious one is that Näätänen et al. (2011) do not include stages for peripheral processing, binaural processing, and grouping. This is understandable, because their model is based on ERP data that are normally obtained with sequential stimuli, eliciting little or no (un)masking and requiring no simultaneous grouping. However, given that Näätänen et al. (2011) also discuss conditions with multiple concurrent auditory streams, this seems to be an omission. Although they also do not address sequential grouping explicitly, this must be implicitly included in the feature detectors and temporal feature recognizers that are part of their model. Another difference between the two models is that Näätänen et al. (2011) view conscious perception as a process acting on items already stored in sensory memory and not as the result of a sequential filtering operation. In that sense, their model resembles that of Cowan (1988), discussed below. This difference is, however, not as fundamental as it seems because the selection process postulated in the current model also can be interpreted as a parallel operation acting on short-term storage (STS); i.e., the preattentive sensory memory can be seen as the part of STS outside the focus of attention. A final distinction between the current model and that of Näätänen et al. (2011) is that the latter directly links most interactions between building blocks with observable ERPs, which makes it more explicit in terms of timing and information flow in the brain. Although most of these links may well be applicable to the current model as well, given the similarity of the two models, they cannot be as specific, because conditions with multiple simultaneous speech signals have hardly been addressed in ERP studies.

Another relevant model is that of Cowan (1988). Although it at first sight seems very different because it is focuses on the role of the memory system while the current model highlights subsequent processing stages, there are several similarities. First, the distinction made by Cowan (1988) between a brief sensory store (up to several hundreds of ms) and a long-term store is reflected in the current model. The latter corresponds to STS; the former also is included but is divided into several preattentive stores with similar short durations. The sensory memory and memory trace involved in MMN generation have, for example, durations of up to 300 ms (Näätänen et al., 2011). Second, Cowan (1988) postulates multiple ways in which attention can be triggered. The occurrence of “gross physical changes in the repeated pattern” is one way, corresponding to bottom-up triggering of attention in the current model. “Voluntary attentional focus” is another way, corresponding to top-down attention modeled here. Third, Cowan (1988) assumes that “perceptual processing occurs independently of attentive processes,” which is consistent with the occurrence of preattentive processing postulated in the current model. There are, however, also differences. Cowan (1988) deliberately bases his model on parallel information processing instead of using a linear time-sequence-based approach. In line with this, he does not interpret selection as a processing stage that either passes through or rejects input, but as a focus placed on items already present in STS. Also, STS itself is not seen as a separate store but as that part of LTM that is activated. As discussed above, this is probably not an essential difference with the current model. Other differences between the current model and that of Cowan (1988) are related to differences in scope and focus between both models and do not reflect fundamental discrepancies. Cowan’s (1988) model, as mentioned earlier, mainly addresses the role of the memory system while the current model focuses on preattentive processing and the operation of attentional control.

## Recommendations for Future Research

Given that the main results of this review have already been summarized in subsections and brought together in a model, I will not present a further overview here but instead focus on recommendations for future research.

1. I think there is an evident need for quantitative models. This review only addresses modeling efforts at the level of peripheral and binaural processing. It appears that a comprehensive model for this level can be derived relatively easily from published models, but that the neurophysiological basis of these models is incomplete. In fact, the only part of most models that has a sound basis is the representation of peripheral critical-band filtering. Quantitative models for the grouping stages have emerged from the work on Computational Auditory Scene Analysis (CASA; see Wang & Brown, 2006). However, because this research mainly aims to optimize separation and analysis of multiple sounds, it is unclear to what degree it generates plausible models of the auditory system. Computational models of attention have mainly been developed for the visual modality (Navalpakkam & Itti, 2005), but some auditory models have been published as well, for example that of Kalinli and Narayanan (2009), which combines acoustic features with lexical and syntactic information to identify stressed syllables in a radio news corpus. Recently, a model covering both grouping and attention was developed by Lutfi, Gilbertson, Heo, Chang, and Stamas (2013). It uses a single factor, derived from statistical differences between sound features (such as F0 and angle of incidence) to predict effects of both target-masker similarity and uncertainty on word perception. Although it is a statistical model, as yet based on relatively few (speech) data, it is of interest because it suggests that the auditory system uses a generic strategy to deal with ambiguous input. While there are, as yet, no integrated models that can quantify early processing and selection of target speech in multi-talker conditions, the combination of several available models developed in different domains would already represent a valuable first step.
2. Research on auditory grouping of speech has up to now mainly focused on effects of voice properties and spatial cues, so that we actually know relatively little about the influence of other speech properties, such as speaking style, linguistic variability, and contextual information. This omission is understandable, because these properties in general affect the intelligibility of the target speech itself, and thus introduce a confound. As demonstrated by Freyman et al. (2001) and Iyer et al. (2010), a simple solution for this problem is to manipulate the properties only in the interfering speech. Another solution would be to vary combinations of properties in the target speech in such a way that the intelligibility does not change.
3. Despite the fact that effects of exogenous auditory attention have emerged in many studies and have been studied extensively in relation to LTM performance, relatively few studies have looked at preconditions for attentional shifts, at the interaction between exogenous and endogenous attention, or at how it affects speech perception in multi-talker conditions. The research of Parmentier and his colleagues, summarized above, represents an exception and a welcome broadening of our knowledge that should be extended by further research.
4. In general, there has not been much research into speech perception where attention is explicitly manipulated while keeping all confounding factors within control. The recent interest in informational masking has generated a number of interesting studies, but the focus on (manipulation of) uncertainty in these studies leaves other important aspects unexplored. Disentangling effects of attention from those of grouping will represent a particular challenge, because these phenomena are easily confounded in behavioral studies. One approach could be to present various pairs of speech stimuli while making sure that each stimulus occurs as target as well as interferer. This would make it possible to separate grouping effects, which depend on pairs, from other effects, depending on individual stimuli.
5. The dynamic properties of attention also deserve further study. Not only should the research into effects of changing voices or locations be extended, but changes in other speech properties should be investigated as well. It would, furthermore, be of great interest to uncover the processes underlying the remarkable sluggishness observed in responses to changes and in adaptation to steady-state conditions.
6. Early behavioral research, such as the study by Cutting (1976) showing that certain dichotic stimuli can at the same time be heard as two sounds and fused into one percept, already indicated that sound perception and sound localization are distinct processes. This is confirmed by more recent electrophysiological and neuroimaging studies revealing separate pathways for analysis of “what” and “where” features in the auditory cortex (Ahveninen, Jääskeläinen, Raij, Bonmassar, Devore et al., 2006; Alain, Arnott, Hevenor, Graham & Grady, 2001). However, there also are indications that the distinction is not clear-cut. The studies by Freyman and collegues, for example, show that perceived spatial location facilitates speech segregation (Freyman et al., 1999). Furthermore, it is evident that binaural perception and sound localization partly rely on the same cues (Middlebrooks & Green, 1991), which indicates that there is an overlap in the processing at peripheral and brainstem levels. This means that it is still not clear to what degree binaural speech perception depends on one’s ability to localize sound target and/or interfering sound sources.

These open issues make it clear that it is unlikely that Cherry’s (1953) paper will soon be forgotten or that the cocktail-party problem will cease to inspire us in the foreseeable future.

## Footnotes

1 Note that these estimations differ from “cloze” probabilities of written text. As shown in Bronkhorst et al. (2002), probabilities of recovering words in sentences measured using orthographic presentation are significantly lower than estimated probabilities for the same material presented auditorily.

2 The actual calculation is more complex, because corrections are applied for hearing loss, high speech levels, and self-masking (upward spread of masking).

3 Binaural MTFs are only calculated for octave bands with center frequencies of 500, 1000 and 2000 Hz. The monaural MTFs are used for the other frequency bands.

4 As discussed below, this also can be interpreted as an attentional effect, namely that listeners find it difficult to focus sustained attention on voice characteristics.

5 In practice, the ILD cannot exceed the bone conduction limit, which is approximately 50 dB (Hood, 1957).

6 Note that this is a unisensory model and that competition between nonauditory and auditory input is not included.
